# Virulence Properties of *mcr*-1-Positive *Escherichia coli* Isolated from Retail Poultry Meat

**DOI:** 10.3390/microorganisms9020308

**Published:** 2021-02-02

**Authors:** Michaela Kubelová, Ivana Koláčková, Tereza Gelbíčová, Martina Florianová, Alžběta Kalová, Renáta Karpíšková

**Affiliations:** Department of Microbiology and Antimicrobial Resistance, Veterinary Research Institute, Hudcova 70, 621 00 Brno, Czech Republic; michicerm@gmail.com (M.K.); gelbicova@vri.cz (T.G.); florianova@vri.cz (M.F.); kalova@vri.cz (A.K.); karpiskova@vri.cz (R.K.)

**Keywords:** APEC, ExPEC, *mcr*-1-positive *E. coli*, WGS, MLST

## Abstract

The great plasticity and diversity of the *Escherichia coli* genome, together with the ubiquitous occurrence, make *E. coli* a bacterium of world-wide concern. Of particular interest are pathogenic strains and strains harboring antimicrobial resistance genes. Overlapping virulence-associated traits between avian-source *E. coli* and human extraintestinal pathogenic *E. coli* (ExPEC) suggest zoonotic potential and safety threat of poultry food products. We analyzed whole-genome sequencing (WGS) data of 46 *mcr*-1-positive *E. coli* strains isolated from retail raw meat purchased in the Czech Republic. The investigated strains were characterized by their phylogroup—B1 (43%), A (30%), D (11%), E (7%), F (4%), B2 (2%), C (2%), MLST type, and serotype. A total of 30 multilocus sequence types (STs), of which ST744 was the most common (11%), were identified, with O8 and O89 as the most prevalent serogroups. Using the VirulenceFinder tool, 3 to 26 virulence genes were detected in the examined strains and a total of 7 (15%) strains met the pathogenic criteria for ExPEC. Four strains were defined as UPEC (9%) and 18 (39%) *E. coli* strains could be classified as APEC. The WGS methods and available on-line tools for their evaluation enable a comprehensive approach to the diagnosis of virulent properties of *E. coli* strains and represent a suitable and comfortable platform for their detection. Our results show that poultry meat may serve as an important reservoir of strains carrying both virulence and antibiotic resistance genes for animal and human populations.

## 1. Introduction

Humans and warm-blooded animals act as natural reservoirs of a wide range of Gram-negative bacteria, such as numerous strains of *Escherichia coli*. Evolutionary processes including rearrangement of the existing genes, their loss or, conversely, acquisition of additional genes, result in the high diversity and plasticity of the *E. coli* genome. Therefore, *E. coli* can be harmless commensal, but also important pathogen harboring both virulence and antimicrobial resistance genes [1,2]. Pathogenic *E. coli* are equipped with a wide range of various virulence factors (VFs), including adhesins, invasins, toxins, and several uptake systems for various nutrients, which enable them to survive in inappropriate conditions (e.g., iron-limited environment in the urinary tract) [3,4].

Intestinal pathogenic *E. coli* can cause mild to life-threatening infections of the gastrointestinal tract in humans and animals. They are equipped with diverse mechanisms of pathogenicity and according to them they may be divided into enterotoxigenic (ETEC), enterohemorrhagic (EHEC), enteroinvasive (EIEC), enteropathogenic (EPEC), and enteroaggregative (EAEC) *E. coli* [2].

Extraintestinal pathogenic *Escherichia coli* (ExPEC), first defined by Johnson and Russo in 2002 [5], have a considerable influence on global health status. Based on the host specificity and preferred site of infection, ExPEC strains are classified into four groups—neonatal meningitis *E. coli* (NMEC), sepsis-associated *E. coli* (SEPEC), uropathogenic *E. coli* (UPEC), and avian pathogenic *E. coli* (APEC). As an extraintestinal pathogen, *E. coli* is the most common Gram-negative bacterium associated with bloodstream infections in both developed and developing countries. UPEC, which can also cause newborn meningitis and sepsis, is the most common cause of community-acquired urinary tract infections (UTIs) [6,7]. Avian pathogenic *E. coli* (APEC), which mainly causes respiratory and systemic disease in poultry, is associated with heavy economic losses in poultry industry [8]. Moreover, based on a large genetic overlap between APEC and certain human ExPEC as well as on numerous experimental studies performed in mammalian and avian animal models, APEC is presumed to have a zoonotic potential and represent an external reservoir for extraintestinal infections in humans [7,9,10].

Colistin, as the last-line drug for the treatment of life-threatening human infections caused by Gram-negative bacteria, is considered as one of the most critically important antimicrobials. It is used in both human and veterinary medicine, including food-producing animals such as poultry [11,12]. Recent studies have described the plasmid-mediated resistance encoded by *mcr* genes, detecting ten different variants of the *mcr* gene of which *mcr*-1 is predominant [13]. The possibility of spreading these genes through global trade with raw meat is being considered in another recent study, with *mcr*-1 gene detected in 19% of turkey meat and liver [14]. Considering previous results, we used the whole-genome sequencing (WGS) data to (i) identify virulence-associated genes in *mcr*-1-positive *E. coli* isolated from retail poultry meat; (ii) assign the tested *E. coli* strains to pathotypes, phylogenetic groups, MLST types and serotypes; and (iii) compare and discuss the results obtained by different APEC diagnostic approaches used in silico and, thus, the virulence properties of *E. coli* strains harboring horizontally transferred genes of antibiotic resistance isolated from retail meat were assessed.

## 2. Materials and Methods

### 2.1. Strain Collection

A total of 46 *mcr*-1-positive *E. coli* strains isolated from retail raw meat collected between March 2017 and January 2019 in the Czech Republic were analysed in this study. Altogether 45 strains were isolated from turkey meat and liver and 1 strain was obtained from chicken liver. Raw meat and liver samples (20 in total) were purchased from the Czech retail market and originated from the Czech Republic (3), Germany (3), Brazil (5), and Poland (9). More details about sample preparation and detection of colistin-resistant bacteria carrying *mcr* genes were described previously [14]. In brief, sample cultivation was performed in buffered peptone water under aerobic conditions at 37 °C overnight. After enrichment, the presence of *mcr*-1 gene was verified by PCR according to Liu et al., 2016 [15]. Positive samples were subsequently inoculated onto Brilliance UTI Clarity agar (Oxoid, Basingstoke, UK) supplemented with colistin sulphate (Discovery Fine Chemicals, Wimborne, UK) at a final concentration of 3.5 mg/L and incubated at 37 °C overnight. Presumptive colonies of *E. coli* (based on colour and colony morphology) were sub-cultured on Blood agar and were identified by MALDI-TOF MS with the use of Biotyper software (version 3.1, Bruker Daltonics GmbH, Bremen, Germany) with a score above 2.0. Up to 5 colonies from each sample were selected for further characterization.

### 2.2. Whole-Genome Sequencing

Genomic DNA was extracted using the Blood and Tissue kit according to the manufacturer’s instructions (Qiagen, Hilden, Germany). The preparation of DNA libraries and sequencing on the Illumina platform were carried out by Eurofins Genomics (Miseq 2 × 300 bp, *n* = 6), Macrogen Korea (Hiseq X^TM^ Ten 2 × 300 bp, *n* = 12), ICM Paris (Nextseq 2 × 150 bp, *n* = 7) and CEITEC VFU (Miseq 2 × 250 bp, *n* = 25). The obtained sequence data was assembled using Velvet version 1.1.04 of Ridom SeqSphere+ (version 3.5.0; Ridom GmbH, Münster, Germany).

### 2.3. Escherichia Genus Strain Phylotyping

Phylogroups were identified by the ClermonTyper at http://clermontyping.iame-research.center/ [16]. The fasta files were downloaded to the above mentioned website. The analysis was accomplished without advanced option.

### 2.4. Multilocus Sequence Typing

*E. coli* sequence type (ST) was determined by the Achtman MLST scheme (www.enterobase.warwick.ac.uk/species/e.coli) [17].

### 2.5. WGS Based Serotyping

Identification of the outer membrane (O) antigen and flagellin protein (H) types was performed using the SerotypeFinder tool from the Centre for Genomic Epidemiology (CGE) website [18].

### 2.6. Virulence Factors Screening

Virulence genes were identified by VirulenceFinder (https://cge.cbs.dtu.dk/services/VirulenceFinder/). Nucleotide sequences of the selected 8 extra genes were downloaded from the NCBI database (Supplementary Materials, Table S1A). The presence of the investigated genes was analysed using Ridom SeqSphere+ software (version 3.5.0; Ridom GmbH, Münster, Germany). Procedure details of the analysis (default settings) were: required identity to the reference sequence—90%, required percentage aligned to the reference sequence—99%. Extra genes were selected according to ExPEC-defining traits, which are described in Johnson et al., 2003 [19]. APEC diagnostic approaches were used as described in Stromberg et al., 2017) [7], Schouler et al., 2012 [20] and Johnson et al., 2008 [21]. Based on criteria by Spurbeck et al., 2012 [22], the strains were defined as UPEC. Criteria of subpathotypes are shown in Table 1.

## 3. Results

### 3.1. Identification of Escherichia coli Phylogroups, Multilocus Sequence Types, Serotypes

From 20 raw poultry meat and liver samples, 46 *mcr*-1 positive *E. coli* strains were selected. These were classified in 7 phylogroups and 30 STs were identified. Phylogenetic analysis revealed that the most of the strains belonged to B1 group 43% (*n* = 20) with 15 unique STs (ST58, ST86, ST156, ST162, ST224, ST453, ST1079, ST1081, ST1167, ST1196, ST1463, ST1582, ST1589, ST2179, ST7973). Fourteen strains were assigned to group A (30%) with 6 different STs (ST10, ST93, ST744, ST746, ST756, ST5956) and to group D (*n* = 5; 11%) within which 5 STs were detected (ST38, ST69, ST349, ST1011, ST7233). Three strains belonged to group E (7%) with ST1140 (*n* = 2) and ST7233 (*n* = 1). Both strains in group F (4%) belonged to ST354, although they were isolated from different samples. In each of groups B2 and C, only 1 strain was detected and the strains belonged to ST1385 (B2) and ST410 (C). Sequence type 7233 was, as the only one, identified in two different phylogroups (D and E).

**Figure 1:**
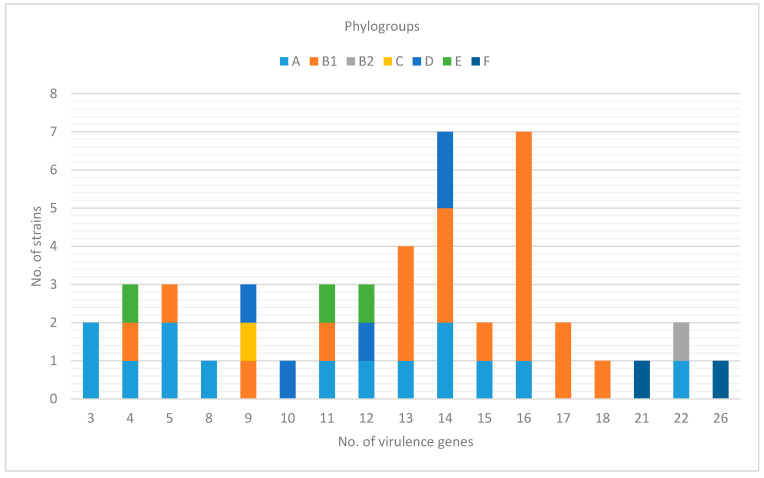
Number of detected virulence genes in *E. coli mcr-1*-positive strains by phylogroups.

O-antigen was identified in 28 (61%) examined strains, whereas serogroups O8 and O89 were the most prevalent (6 and 4 strains). In 13 strains (28%) the O-antigen was not identified. Detailed results are shown in Figure 1 and Supplementary Materials, Table S1B.

### 3.2. Virulence Genes Screening

**Figure 2:**
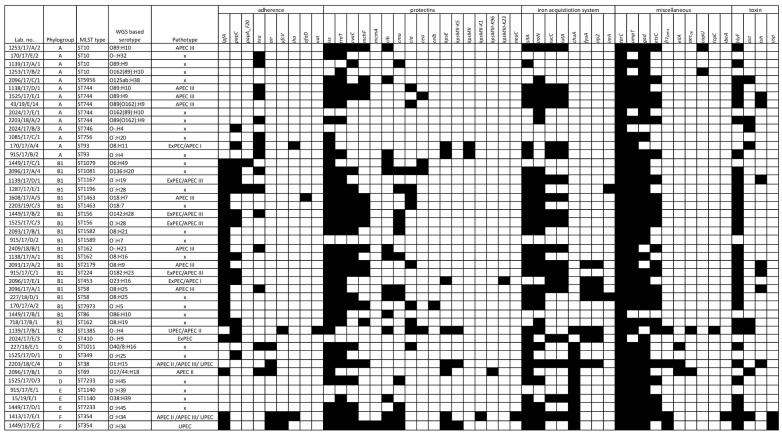
Characteristics of 46 tested *E. coli* strains including phylogroup, MLST type, serotype, and virulence genes presence. APEC—avian pathogenic *E. coli* based on criteria by Stromberg et al., 2017 [7] (APEC I); by Schouler et al., 2012 [20] (APECII), by Johnson et al., 2008 [21] (APECIII); ExPEC—extraintestinal *E. coli*; UPEC—uropathogenic *E. coli*; ×—non-pathogenic *E. coli;* Black boxes represent presence and white boxes absence of respective gene.

From 3 to 26 virulence genes (VGs) were confirmed in the tested strains (Figure 2; Table S1B). Based on the established criteria of pathotype definition (Table 1), 7 (15%) strains possessed ExPEC characteristics, 4 were defined as UPEC (9%), and 18 (39%) as APEC. Determination of APEC strains differed based on selected criteria. Using the Johnson et al., 2008 [21] approach, 14 strains were recognized as APEC, whereas only 4 strains met APEC criteria using Schouler et al., 2012 [20] method and only 3 strains harbored APEC virulence genes described by Stromberg et al., 2017 [7]. Two strains were confirmed as APEC by 2 different diagnostic approaches simultaneously.

Altogether 26 (56%) strains did not fully comply with the characteristics of the selected pathotypes, but they still carried some specific virulence genes (Figure 1, Supplementary Table S1B). Out of these strains, 15 harbored 4 virulence genes detected according to the Johnson et al., 2008 [21] APEC diagnostic approach.

A total of eight *E. coli* strains were found to be positive for the presence of some *kps* genes (K2 capsule), of which one strain carried *ibeA*—gene encoding invasin responsible for neonatal meningitis in humans [23,24]. This *E. coli* strain (Lab. no. 1413/17/E/1) was isolated from turkey meat originating in the Czech Republic.

None of the tested strains contained specific virulence genes relevant for intestinal *E. coli* pathotypes, except of eight strains harboring heat-stable enterotoxin *astA*.

## 4. Discussion

The strains used in this study were chosen from the set of *mcr*-1-positive strains isolated from retail raw meat products [14]. Since the emergence of the plasmid-mediated colistin (CT) resistance encoded by the *mcr*-1 gene in Chinese animal production [15], the worldwide dissemination of this CT resistance has been recorded [25]. The occurrence of Enterobacteriaceae with the *mcr*-1 gene has been reported in the human population in connection with animal products from different animal species [26,27]. Both of these facts and the role of colistin as the last treatment resort in serious human infections caused by multidrug-resistant bacteria should lead to a great concern around the world [28]. The resistance to antimicrobials can be based on DNA mutation or on horizontal gene transfer. Many genes, coding for resistance to antimicrobials, are inserted into conjugative plasmids, transposons or integrons, called mobile genetic elements. These elements may also carry virulence factor determinants and, therefore, a correlation between virulence and antimicrobial resistance has been documented, at least in some *E. coli* clones [2].

Virulence potential of *E. coli* is determined by the occurrence of virulence genes, coding for colonizing factors (fimbriae and adhesins), survival of bacterial cells in unfavorable environments (protectins and siderophores) or causing the host inflammatory response, e. g. toxin production [2]. In the past, different systems have been used to assess the virulence potential of *E. coli*. Based on recent knowledge, determination of serotypes is not satisfactory, even if some of them are more frequently associated with certain infections than others [29,30]. Out of the serogroups, described as typical of APEC, only O1 (*n* = 1), O18 (*n* = 2) and O8 (*n* = 7) were detected, whereas APEC virulence genes were only confirmed in five of these strains. In the rest of APEC strains, either atypical serogroups O17, O23, O89 and O182 were confirmed or the serogroup was not identified (O-; 28%). Therefore, our findings support the opinion that the association between serogroup and virulence potential is weak.

Phylotyping is a simple method for the assessment of clinical significance of *E. coli* strains. Based on the presence of four different genes or DNA fragments, *E. coli* can be classified into eight phylogroups A, B1, B2, C, D, E, F, or cryptic clade I [31]. It has been confirmed that commensal strains mostly fall into A and B1 phylogroups. Intestinal pathogenic *E. coli* affiliate to groups A, B1, or D. The most of the ExPEC strains belong to groups B2 and D, whereas human pathogenic strains are usually clustered in group B2 [32]. The strains from phylogroup C are considered to be a sister group to those in B1, group E is related to group D and phylogroup F is very similar to B2 [24,33]. In our study, all strains belonging to the phylogroups B2 (*n* = 1), D (*n* = 2), and F (*n* = 2), which are associated with highly virulent *E. coli* pathotypes [31,34], were determined as pathogenic based on selected criteria in contrast to strains from phylogroup E (3), which did not fully meet the definition of selected pathotypes. On the contrary, the majority of pathogenic strains (9/20; 45%) belonged to B1 or A phylogroup (5/20; 25%). Phylogenetic analysis of all 46 tested strains revealed that the most of *E. coli* strains belonged to B1 (43%), followed by groups A (30%), D (11%), E (7%), F (4%), B2 (2%), and C (2%). Our data are in accordance with results obtained for 409 *E. coli* strains from commercial chicken carcasses in Brazil where the most frequent phylogroup was B1 (36.6%), followed by A (31.7%), D (28.1%), and B2 (3.40%) [35]. Other studies aimed at characterization of *E. coli* isolates from retail poultry meat and eggs described B2 and D as the major phylotypes in ExPEC, while non-ExPEC isolates belonged to groups A and B1 [34,36].

The MLST scheme is another method that is used for *E. coli* typing. It provides good means for *E. coli* typing, but there is no direct correlation between STs and their phylogroup affiliation [37]. In our study, 30 different ST types were identified. Out of them, 22 were unique, which supports the finding that *E. coli* are highly diverse (Figure 1). The most prevalent was ST744 (11%). All these strains were assigned to group A, O89, but were isolated from different samples originating in different countries (Poland, Germany, and Brazil). In three samples, ST162 was detected, which was described by Zhuge et al., 2019 [28] as a highly virulent *mcr*-positive *E. coli* clone. Only one of these strains (Lab. no. 2409/18/B/1) fully met the characteristics of APEC by the Johnson et al., 2008 [21] approach.

The developed PCR and sequencing techniques, especially whole genome sequencing methods, enable detailed screening of the occurrence of specific genes and open new possibilities to estimate the relationship between the presence of genes and pathogenicity of *E. coli* strains [29,38]. The VirulenceFinder software has been updated in 2020 and a broader spectrum of virulence genes encoding, e.g., adhesins, siderophores, toxins, and protectins have been included in multiple variants [39]. Therefore, at least three virulence genes were detected in the tested strains. Genes affecting resistance to host innate immunity (e.g., *ompT*) or protecting against phagocytosis (e.g., *iss*) have often been detected, followed by genes employed in the iron acquisition system or transport system, e.g., *sitA* gene encoding periplasmic-binding protein and *iroN* encoding the outer membrane receptor fepA belonging to ExPEC siderophores group or *iutA* gene.

Using WGS data, we focused on the identification of genes by two approaches, which are routinely employed in our laboratory for APEC detection in vitro [20,21]. The method according to Schouler et al., 2012 [20] is based on extensive characterization of 1491 avian *E. coli* isolates from France, Spain and Belgium. We detected two out of four patterns of VGs-B (*iutA*+ P(F11)− *frz_orf4_+*) and D (*iutA*− *sitA+ aec26*+) in four samples from our collection of strains. The method takes into account different strategies of *E. coli* to invade the host according to genetic equipment of bacterial strain and uses both chromosomally and plasmid located VGs. On the other hand, to identify traits that predict APEC virulence, five plasmid pathogenicity-associated island (PAI) genes were validated for rapid diagnostics according to Johnson et al., 2008 [21]. Fourteen of 46 *E. coli* isolates carried all of five genes associated with highly pathogenic APEC strains (*iutA*, *hlyF*, *iss*, *iroN*, *ompT*). However, four out of five VGs were detected in 37% of strains and three VGs were detected in 4% of strains. The relationship between in vivo virulence and the number of PAI genes in one-day-old chicks was analysed by de Oliveira et al., 2015 [40], who found that 95% of APEC isolates harbored the two, three, or four above mentioned genes, even though isolates with fewer than two VGs were rarely pathogenic. Taking into account these results, the occurrence of APEC strains in our study increased up to 72% (33/46). However, the study of de Oliveira et al., 2015 [40], also showed that two or more VGs are found in approximately 50% of the non-pathogenic isolates. These results suggested that the presence of two or more VGs is necessary but not sufficient to turn *E. coli* into APEC and, therefore, such interpretation is uncertain.

## 5. Conclusions

Based on various MLST types, serotypes, and virulence genes detected, high diversity of examined strains was confirmed. Our results show that *E. coli* strains with plasmid mediated colistin resistance isolated from poultry meat products disposed of various virulence genes. In total, 43% of these strains were assigned to at least one of the assessed pathotype (APEC, 39%; ExPEC, 15%; UPEC, 9%) and may pose a threat of further spreading to the environment, animals, and humans, especially if the hypothesis is taken into account that a combination of resistance and virulence properties may be a kind of advantage for *E. coli* under a selective pressure of antimicrobials.

## Figures and Tables

**Table 1:** Criteria of ExPEC subpathotypes.

Pathotypes	Gene Content	Virulence Genes (VGs)	Function	References
ExPEC	≥2 VGs	*papA/papC*	Adhesion	Johnson et al., (2003) [19]
		*sfa/foc*	Adhesion	
		*afa/dra*	Adhesion	
		*kpsM II*	Protectins	
		*iutA*	Iron uptake	
UPEC	≥2 VGs	chuA	Iron uptake	Spurbeck et al., (2012) [22]
		fyuA	Iron uptake	
		*vat*	Toxin	
		*yfcV*	Adhesion	
APEC I	ExPEC plus ≥4 VGs	*kpsM II*	Protectins	Stromberg et al., (2017) [7]
		*iss*	Protectins	
		*tsh*	Toxins	
		*iutA/fyuA*	Iron uptake	
		*sfa/foc/papA/papC/papEF*	Adhesion	
APEC II	pattern A: *iutA* + P(F11)	*iutA*	Iron uptake	Schouler et al., (2012) [20]
	pattern B: *iutA* + *frz_orf4_*	P(F11) ^a^	Adhesion	
	pattern C: *iutA* + O78	*frz_orf4_*	Sugar transport system	
	pattern D: *sitA* + *aec26*	*aec_26_*	Type VI secretion system	
		*sitA*	Iron uptake	
APEC III	5 VGs	*iutA*	Iron uptake	Johnson et al., (2008) [21]
		*iss*	Protectins	
		*hlyF*	Toxins	
		*iroN*	Iron uptake	
		*ompT*	Aspartyl protease	

ExPEC—extraintestinal *E. coli*, APEC—avian pathogenic *E. coli*, UPEC—uropathogenic *E. coli*. ^a^ strains were noted as P(F11)+ when combination of genes—*felA*, *papC*, and a variant of *papG* were present.

## Data Availability

The data for this study have been deposited in the European Nucleotide Archive (ENA) at EMBL-EBI under accession number PRJEB34874 (https://www.ebi.ac.uk/ena/data/view/PRJEB34874).

## Supplementary Materials

The following are available online at https://www.mdpi.com/2076-2607/9/2/308/s1, Table S1: Accession number of genes selected for ExPEC genes screening and detail results of virulence genes findings.
